# Paternal genetic background orchestrates fetal development through tissue-specific *cis*-regulatory divergence

**DOI:** 10.1016/j.isci.2026.116684

**Published:** 2026-07-13

**Authors:** Muhammad Arsalan Iqbal, Eduard Murani, Frieder Hadlich, Henry Reyer, Michael Oster, Nares Trakooljul, Klaus Wimmers, Siriluck Ponsuksili

**Affiliations:** 1 Research Institute for Farm Animal Biology (FBN), Wilhelm-Stahl-Allee 2, 18196 Dummerstorf, Germany; 2 University Rostock, Faculty of Agricultural and Environmental Sciences, 18059 Rostock, Germany

**Keywords:** allele-specific expression, ASE, fetal transcriptional programming, pig, tissue-specific ASE, paternal genetics

## Abstract

Paternal influences on offspring development extend beyond Mendelian inheritance, but how sire genetics shape fetal transcription and *cis*-regulatory landscapes across tissues is unclear. Using a reciprocal backcross in pigs (F_1_×German Landrace vs. F_1_×Piétrain) and a haplotype-agnostic RNA-seq pipeline, we profiled six fetal tissues to test sire-breed effects. We identified paternal breed-driven transcriptional divergence, with 1,176 genes differentially expressed. Transcriptional divergence was most pronounced in metabolic tissues, revealing a tissue-specific trade-off: F_1_ × GL conceptuses prioritized metabolic and energy pathways, whereas F_1_ × Pi conceptuses upregulated developmental and cell cycle processes. Allele-specific expression analysis revealed extensive, tissue-dependent *cis*-regulatory divergence, and differential ASE highlighted candidate mediators, including *SLC5A12* and *IVD* (kidney), *BRD3* (brain), and *CES1* (liver). These results indicate that paternal genetics programs fetal tissues via *cis*-regulatory variation, informing strategies to improve livestock traits. Notably, many loci overlapped known QTLs, suggesting that regulatory variants connect sire background to performance-related phenotypes and fetal programming.

## Highlights

• Sire’s genetic background drives tissue-specific trade-offs in fetal programming
• GL-sired conceptuses upregulate oxidative and energy metabolism pathways
• Pi-sired conceptuses enhance developmental plasticity and proliferation
• Differential allelic expression reveals sire-dependent *cis*-regulatory divergence

## Graphical abstract

**Figure:**
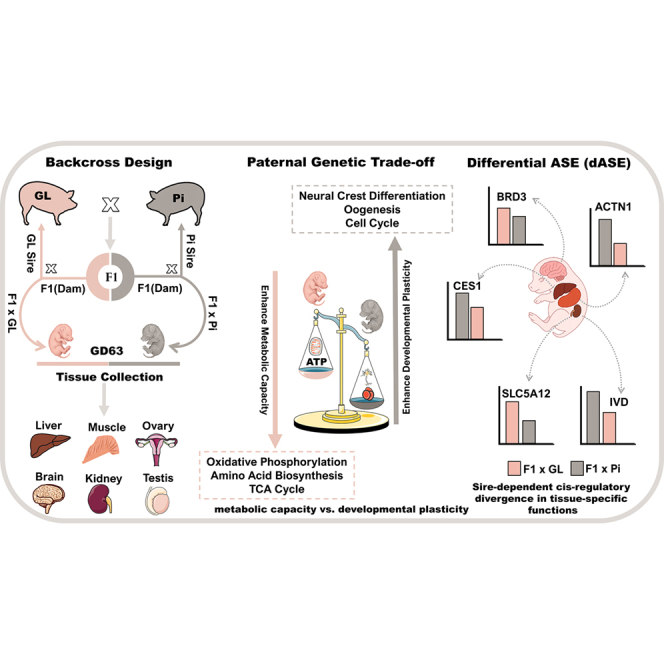

## Teaser

Genomics; Developmental biology; Livestock husbandry

## Introduction

Genetic improvement in livestock relies on understanding how parental genomes regulate fetal development, a critical period for programming postnatal growth, metabolism, and reproduction.^1^ Disruptions during gestation, such as intrauterine growth restriction, impose permanent consequences on neonatal viability through complex genetic-epigenetic-environmental interactions.^2^^,^^3^^,^^4^^,^^5^^,^^6^ While genomic imprinting is a well-established mechanism of parent-of-origin-specific regulation, with over 180 loci characterized in humans and mice,^7^^,^^8^ it remains poorly defined in pigs, with only ∼20 genes experimentally validated.^8^^,^^9^ Furthermore, recent evidence suggests that epigenetic mechanisms in development are often driven by fetal genetics itself, underscoring a significant and direct role for inherited paternal alleles in developmental programming beyond canonical imprinting.^10^

Allele-specific expression (ASE) analysis provides a powerful, high-resolution tool to detect *cis*-regulatory divergence by measuring the unequal transcription of parental alleles in heterozygous individuals.^11^ Unlike expression quantitative trait locus (eQTL) mapping, which requires large cohorts and lacks individual allelic resolution, ASE can uncover *cis*-regulatory variation within individuals, making it ideal for studies with limited sample sizes.^12^^,^^13^ This approach has uncovered genome-wide *cis*-regulatory effects across diverse model organisms, from Drosophila and mice to humans,^14^^,^^15^^,^^16^ and documented parent-of-origin biases in livestock, including cattle and pigs.^17^^,^^18^^,^^19^^,^^20^ In livestock, reciprocal crosses have revealed extensive parent-of-origin-biased gene expression, including a marked predominance of paternal alleles in cattle muscle and liver,^18^ as well as in postnatal porcine brain^19^ and mid-gestation muscle tissue.^20^ These studies confirm ASE as a conserved mechanism for parent-biased and breed-specific regulation.

Despite these advances, a critical gap remains: the effects of paternal genetic background on ASE during fetal organogenesis are largely uncharacterized. The mid-gestation period represents a critical developmental window in pigs, during which over 70% of skeletal muscle fibers are formed,^21^ neural tube closure and key neurodevelopmental processes are completed,^22^ and gonadal differentiation initiates long-term reproductive programming.^23^ This makes day 63 a critical window for investigating how paternal genetics influences the establishment of tissue-specific gene regulatory networks. Shifts in allelic expression during this period could therefore determine lifelong metabolic and physiological patterns. The genetically divergent German Landrace (GL) and Piétrain (Pi) breeds, selected for maternal traits and muscularity/leanness, respectively,^24^^,^^25^ provide an ideal model to investigate this paternal influence on early-stage epigenetic and transcriptional control.

Understanding how sire genetics shape fetal programming extends beyond livestock breeding to address fundamental questions in reproductive biology. While maternal contributions to fetal development are well-documented,^2^^,^^6^ paternal alleles can exert distinct, tissue-specific effects through mechanisms that remain incompletely characterized.^1^^,^^10^ Characterizing these paternal influences is therefore crucial for advancing fundamental knowledge of reproductive biology and for informing livestock management strategies that aim to improve offspring viability, growth, and long-term performance.^26^

A core limitation of conventional ASE analysis in livestock is its dependence on phased parental haplotypes, making it impractical in studies lacking fully characterized parental genomes.^27^^,^^28^ To overcome this, we implemented a robust, well-established RNA-seq variant discovery pipeline that reliably detects regulatory variants with or without haplotype information.^29^^,^^30^^,^^31^ Specifically, we adopted a comprehensive framework that includes: accurate SNP/indel detection via GATK Best Practices^32^; reference bias correction through WASP filtering enhanced by N-masking of known variants^33^^,^^34^; and statistical inference of ASE using ASEP, enabling *cis*-regulatory discovery without reliance on haplotype phasing.^35^

Within a reciprocal backcross design (F_1_ sows × GL or Pi sires),^26^ we applied this framework to profile six fetal tissues at day 63 of gestation. Our study aimed to (i) characterize transcriptomic differences both between backcross groups (F_1_ × GL and F_1_ × Pi) and across tissues; (ii) map tissue-specific ASE landscapes within each backcross group; and (iii) identify differential ASE (dASE) genes by comparing ASE between reciprocal backcrosses within each tissue. By integrating bulk and allele-specific transcriptional analyses within a controlled reciprocal cross, this study provides unprecedented insight into how paternal genetic variation shapes fetal programming through regulatory mechanisms, directly connecting *cis*-regulatory divergence in fetal tissues to the genomic architecture of complex production traits. Importantly, our haplotype-agnostic ASE framework extends beyond livestock systems, offering a conceptual and analytical model for investigating paternal regulatory inheritance in mammals. These insights advance reproductive biology at a fundamental level while also providing a mechanistic foundation for precision breeding and evidence-based sire selection strategies to improve offspring growth, viability, and long-term productivity.

## Results

**Figure 1:**
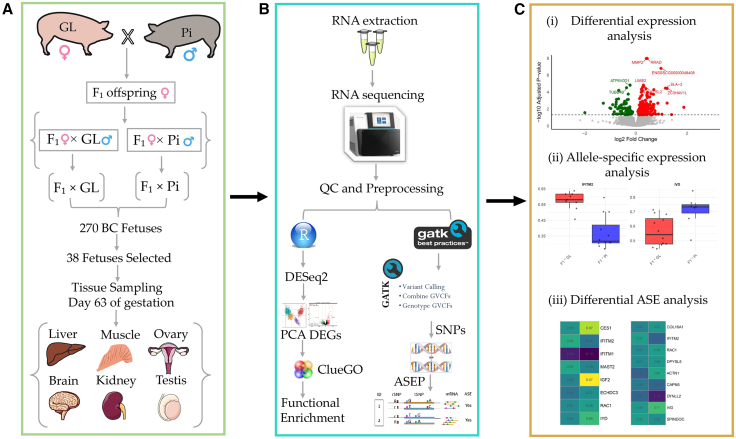
Schematic overview of the experimental design and bioinformatic workflow (A) Paternal lines (GL and Pi) were crossed to generate F1 offspring, followed by reciprocal backcrosses (F_1_ × GL and F_1_ × Pi). At gestational day 63, tissues (liver, muscle, ovary, brain, kidney, and testis) were collected from 38 selected backcross fetuses (for details on tissue sampling, see Methods section 2.1). (B) RNA was extracted from six fetal tissues, sequenced, and subjected to quality control and preprocessing. The processed data were used for (i) differential expression analysis (DESeq2) and functional annotation (ClueGO) and (ii) SNP discovery using the GATK pipeline, with subsequent allele-specific expression analysis (ASEP). (C) Summary of analysis results: (i) DEGs, (ii) ASE patterns, and (iii) differentially regulated ASE events.

The mRNA sequencing data for this study were generated from a subset of 38 fetal samples from F_1_ × GL and F_1_ × Pi backcrosses, collected at day 63 of gestation. Six tissues were analyzed: liver, brain, muscle, kidney, ovary, and testis (Figures 1A and 1B). In total, 3.95 billion raw reads from 131 libraries were generated, with 3.42 billion successfully mapped to the Sscrofa11.1 reference genome, yielding an average mapping rate of approximately 86.5% (Table S1). To extract transcriptomic insights from the RNA-seq data, three complementary analytical approaches were applied: (i) differential gene expression analysis was used to compare transcript abundance between backcross groups within each tissue, based on tissue-specific gene sets filtered for adequate expression (≥5 counts in at least 80% of samples per tissue; see Methods 2.3); (ii) ASE analysis was carried out separately for each tissue and backcross group using 87,484 high-confidence heterozygous SNPs commonly identified across samples, and (iii) dASE analysis assessed allelic imbalance between the two groups at the gene level by aggregating SNP-level ASE data for genes containing at least three heterozygous SNPs, resulting in 7,126 genes tested per tissue (Figures 1B and 1C).

### Phenotypic weight distribution and genotypic clustering by backcross group

**Figure 2:**
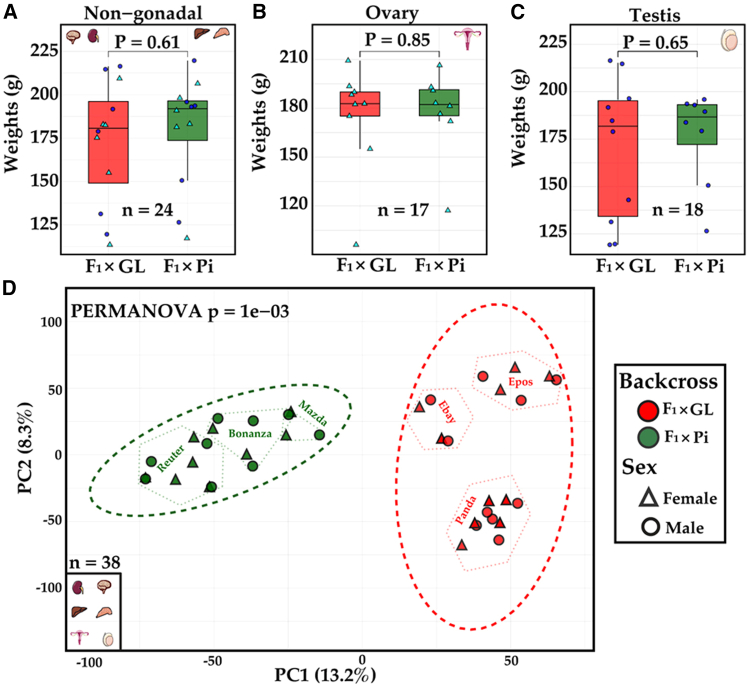
Fetal weight distribution and genotype structure by backcross (A–C) Fetal weights at gestational day 63 for (A) non-gonadal tissues, (B) ovary, and (C) testis, comparing F_1_ × GL and F_1_ × Pi backcrosses. The red boxplots represent F_1_ × GL; dark-green boxplots represent F_1_ × Pi. Boxplots show median and interquartile range; individual fetuses are shown as points. Sample sizes (*n*) and two-sided *t* test *p* values are shown in each figure. (D) PCA of SNP-chip genotypes (*n* = 38) showing genetic separation between F_1_ × GL and F_1_ × Pi. Points are colored by backcross, and dashed ellipses represent 95% confidence intervals for group centroids (PERMANOVA *p* = 1 × 10^–3^). Percent variance explained by each principal component is indicated on the axes. Across all figures, sex is indicated by shape (triangle = female; circle = male). Semi-transparent, sire-specific partitions indicate the distribution of offspring from each sire within the two backcross groups (light red, F_1_ × GL; light green, F_1_ × Pi). The F_1_ × Pi backcross includes the sires Reuter, Bonanza, and Mazda, while the F_1_ × GL backcross includes Epos, Ebay, and Panda.

We first assessed phenotypic and genotypic differences between conceptuses from the two paternal breeds. Analysis of fetal weights at gestational day 63 revealed no significant differences between backcross groups. Based on the available fetal tissues, slightly different group compositions were analyzed. For non-gonadal comparisons, a matched set of 24 fetuses was used (*n* = 12 per backcross group). Mean fetal weights did not differ significantly between the F_1_ × GL (172.6 ± 35.6 g) and F_1_ × Pi (179.7 ± 31.6 g) groups (*p* = 0.609; Figure 2A). For gonadal tissues, sample numbers varied depending on tissue availability, with 17 ovaries (F_1_ × GL: *n* = 9; F_1_ × Pi: *n* = 8) and 18 testes (F_1_ × GL: *n* = 10; F_1_ × Pi: *n* = 8). The mean fetal weight in the cohort used for the analysis of ovaries was 174.7 ± 32.9 g (F_1_ × GL) and 185.2 ± 26.7 g (F_1_ × Pi) (*p* = 0.847; Figure 2B). In the cohort used for the analysis of testes, mean weights were 169.6 ± 38.0 g (F_1_ × GL) and 176.5 ± 24.9 g (F_1_ × Pi) (*p* = 0.647; Figure 2C). In contrast to the homogeneous weight distribution, principal component analysis (PCA) of SNP chip genotypes revealed clear genetic structure corresponding to the experimental pedigree (Figure 2D). The primary axis of variation (PC1, 13.2% total variance explained) captured significant backcross group divergence. PERMANOVA on the Euclidean distance matrix confirmed that the backcross group explained 12.5% of total genetic variance (R^2^ = 0.125, *p* = 0.001). Further supporting this, univariate analysis revealed that 90.4% of variation along PC1 was attributable to backcross group (p < 2e-16), demonstrating that PC1 effectively separates F_1_ × GL from F_1_ × Pi conceptuses. The secondary axis (PC2, 8.3% total variance explained) captured within-group genetic structure, dominated by individual sire identities (Figure 2D). Within-breed analyses confirmed strong sire effects in both German Landrace (94.9% variance explained, *p* < 0.001) and Piétrain (53.4%, *p* = 0.003) lines. In contrast, biological factors, including fetal sex or fetal weight, showed no significant association. These results demonstrate that PC1 captures the intended breed-level genetic divergence, while PC2 reflects the expected genetic relatedness among fetuses sharing parents, validating the genetic structure of our reciprocal backcross design.

### Principal component analysis of global and tissue-specific clustering

**Figure 3:**
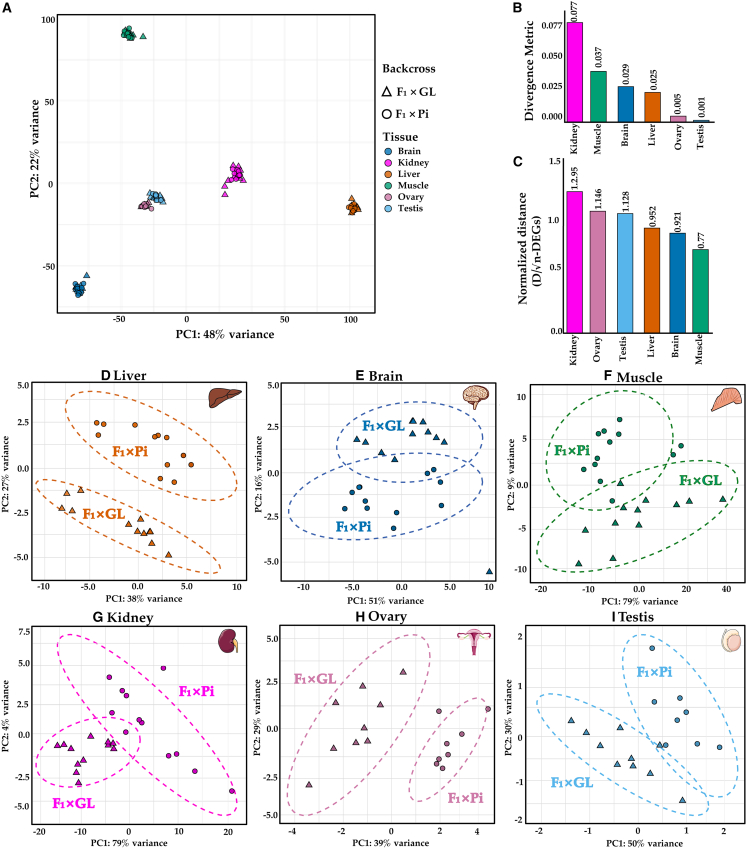
Global and tissue-specific transcriptome divergence across backcross groups (A) Global PCA on VST counts from all samples, visualizing major sources of variation across tissues and backcross groups. Percent variance explained by PC1 and PC2 is indicated on the axes. (B) The bar chart depicts transcriptome-wide divergence between backcross groups quantified using a DEG-based divergence score (Δ), calculated as the mean squared difference in group-average expression normalized by the number of DEGs. (C) The bar chart represents the normalized Euclidean distance between backcross-group centroids in PCA space. Distances were calculated using the first two principal components (PC1 and PC2) and normalized by √(n_DEGs) to account for differences in the number of DEGs across tissues. (D–I) Tissue-specific PCAs using DEGs identified between backcross groups: (D) liver, (E) brain, (F) muscle, (G) kidney, (H) ovary, and (I) testis, with the percent variance explained labeled on both axes. Colors and shapes are consistent across figures: liver = orange, brain = blue, muscle = green, kidney = pink, ovary = magenta, testis = light blue; triangle = F_1_ × GL, circle = F_1_ × Pi. Dashed ellipses represent 95% confidence intervals for each tissue-backcross group combination.

Principal component analysis (PCA) of transcriptomic data was performed at two levels. A global PCA, using all tissues and backcross groups, revealed that samples clustered primarily by tissue type (PC1: 48%, PC2: 22%; total: 70% variance). Reproductive tissues (ovary and testis) clustered together, indicating a shared transcriptomic profile distinct from other tissues (Figure 3A). The global PCA was performed on variance-stabilizing transformed (VST) counts to visualize the dominant sources of biological variation. For tissue-specific differential expression analyses, we adjusted for multiple covariates to ensure that separation between backcross groups reflects biological differences rather than unwanted effects. Transcriptomic divergence was quantified using a differentially expressed gene (DEG)-based divergence score (Δ), with values for each tissue as follows: kidney (Δ = 0.077), muscle (Δ = 0.037), brain (Δ = 0.029), liver (Δ = 0.025), ovary (Δ = 0.005), and testis (Δ = 0.001) (Figure 3B). To enable cross-tissue comparison, we computed a normalized divergence metric (centroid distance divided by the square root of the number of tissue-specific DEGs). This normalized metric revealed the strongest paternal breed-driven divergence in kidney (normalized distance = 1.295), followed by ovary (1.146), testis (1.128), liver (0.952), brain (0.921), and muscle (0.77) (Figure 3C). For the PCA shown for tissue-specific DEGs, we used VST values restricted to the DEG set across all samples for visualization. PCA of DEG expression profiles revealed distinct patterns of tissue-specific transcriptomic divergence between backcross groups (Figures 3D–3I). Kidney and muscle showed the clearest separation along PC1, which accounted for 79% of the variance in each tissue (Figures 3F and 3G). Liver also showed visible separation between groups (PC1: 38%; Figure 3D). In the brain, PC1 explained 51% of the variance (Figure 3E). In the ovary and testis, PC1 explained 39% and 50% of the variance, respectively (Figures 3H and 3I).

### Global differential expression and functional enrichment analysis across fetal tissues

**Figure 4:**
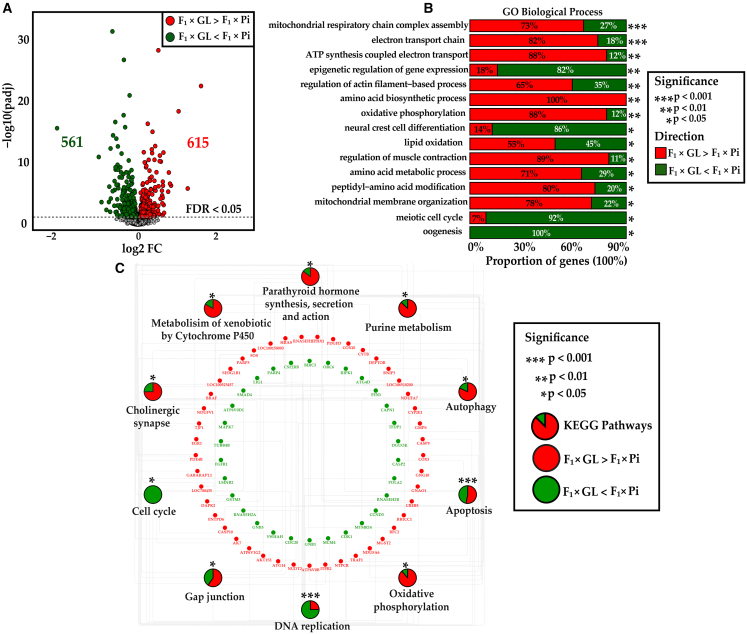
Differential expression and functional enrichment of genes between backcross groups (A) Volcano plot indicated the DEGs between the backcross groups across fetal tissues. (B) Stacked bar plot depicting gene ontology (GO) biological process enrichment of DEGs; bar length represents the proportion of enriched genes. (C) KEGG pathway enrichment network of DEGs. Pie charts indicate the proportion of genes enriched in each pathway. Consistent color coding was applied across all figures; upregulated genes (F_1_ × GL > F_1_ × Pi) are shown in red, and downregulated genes (F_1_ × GL < F_1_ × Pi) are shown in dark green. Statistical significance is denoted by asterisks (∗*p* < 0.05, ∗∗*p* < 0.01, ∗∗∗*p* < 0.001).

Global differential expression analysis across six fetal tissues was performed to identify genes differing between backcross groups (F_1_ × GL vs. F_1_ × Pi). In total, 1,176 DEGs (FDR <0.05) were identified, of which 615 were upregulated and 561 downregulated in F_1_ × GL relative to F_1_ × Pi (Figure 4A; Table S2). Functional enrichment analysis revealed that upregulated genes in F_1_ × GL were strongly associated with mitochondrial energy production and biosynthesis, including the amino acid biosynthetic process (100% of term genes, *p* ≤ 0.01), electron transport chain (82%, *p* ≤ 0.001), and oxidative phosphorylation (88%, *p* ≤ 0.01; Figure 4B). KEGG pathway analysis further confirmed enhanced energy metabolism in F_1_ × GL, with significant enrichment of purine metabolism (88% of pathway genes upregulated, *p* ≤ 0.05) and autophagy (82%, *p* ≤ 0.05) (Figure 4B; Table S3). In contrast, developmental and regulatory processes were predominantly downregulated in F_1_ × GL, while upregulated in F_1_ × Pi, including oogenesis (100% of term genes, *p* ≤ 0.05), neural crest cell differentiation (86%, *p* ≤ 0.05), and epigenetic regulation (82%, *p* ≤ 0.01). The KEGG analysis further revealed preferential suppression of cell cycle progression in F_1_ × GL, with cell cycle (100% downregulated, *p* ≤ 0.05) and DNA replication (75% downregulated, *p* ≤ 0.001) pathways enriched in F_1_ × Pi (Figures 4B and 4C).

### Tissue-specific differential expression and functional enrichment between backcross groups

**Figure 5:**
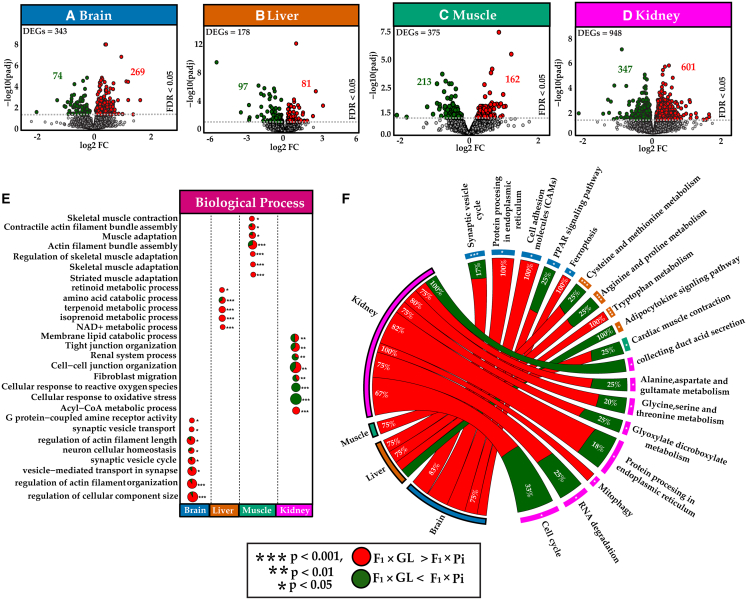
Tissue-specific DEGs and functional enrichment between backcross groups (A–D) Volcano plots showing the number and distribution of DEGs in (A) brain, (B) liver, (C) muscle, and (D) kidney between F_1_ × GL and F_1_ × Pi reciprocal backcrosses. (E) Pie chart-style dot plot illustrating gene ontology (biological process) enrichment. The size of each pie reflects the total number of genes in the enriched term, and the proportion of each pie indicates the fraction of genes upregulated in F_1_ × GL (red) versus F_1_ × Pi (dark green). (F) Chord diagram showing KEGG pathway enrichment. The width of each chord reflects the gene count within the pathway, with red segments representing genes upregulated in F_1_ × GL and dark green segments representing genes upregulated in F_1_ × Pi. Statistical significance is denoted by asterisks (∗*p* < 0.05, ∗∗*p* < 0.01, ∗∗∗*p* < 0.001).

To investigate tissue-specific regulatory divergence between backcross groups, we performed differential expression analysis within six fetal tissues (brain, liver, muscle, kidney, testis, and ovary) between F_1_ × GL and F_1_ × Pi reciprocal backcrosses. We identified tissue-specific DEGs (FDR <0.05), with the kidney exhibiting the highest divergence (948 DEGs), followed by muscle (375), brain (343), and liver (178; Figures 5A–5D). Reproductive tissues showed fewer DEGs, with 43 in the testis and 11 in the ovary (Table S4).

DEGs were partitioned into distinct expression clusters. Cluster 1 was enriched for genes upregulated in F_1_ × GL relative to F_1_ × Pi, while Cluster 2 was enriched for genes downregulated in F_1_ × GL (as described in Methods section 2.8). Subsequent tissue-specific enrichment analyses revealed distinct functional profiles for the two backcrosses (Table S5). In the brain, genes upregulated in F_1_ × GL showed robust enrichment for neural and synaptic functions. Significantly overrepresented terms included cytoskeletal and actin filament organization, vesicle-mediated transport, synaptic vesicle processes, and G protein-coupled amine receptor activity. Corresponding KEGG pathways included the synaptic vesicle cycle, protein processing in the endoplasmic reticulum, cell adhesion molecules, PPAR signaling, and ferroptosis. In contrast, genes upregulated in the F_1_ × Pi did not yield strong or coherent neural pathway enrichments (Figures 5E and 5F).

In the liver, metabolic trade-offs were observed: the genes upregulated in F_1_ × GL were enriched for processes including NAD^+^ metabolism, along with isoprenoid, terpenoid, and retinoid biosynthesis. Conversely, genes upregulated in F_1_ × Pi were associated with adipocytokine signaling and specific amino acid metabolic pathways. Several amino acid metabolism terms exhibited mixed-direction regulation, suggesting partial bidirectional control between the backcrosses. In muscle, genes upregulated in F_1_ × GL were predominantly associated with core pathways of muscle structure and function, including skeletal muscle contraction, muscle adaptation, and the assembly of contractile actin filament bundles. Genes upregulated in F_1_ × Pi were more evenly distributed across various metabolic and regulatory terms and did not predominantly enrich these canonical contractile pathways (Figures 5E and 5F).

In the kidney, the enrichment profiles suggested complementary physiological roles. Genes upregulated in F_1_ × GL were enriched for acyl-CoA metabolism and ER protein processing. Genes upregulated in F_1_ × Pi were associated with oxidative stress responses, collecting duct acid secretion, and renal developmental processes. In reproductive tissues, a limited number of DEGs were identified, and no significant pathway-level enrichments were detected for either backcross (Figures 5E and 5F). Full enrichment statistics, gene counts, and the proportions of up- and downregulated genes within each enriched pathway and biological process are provided in Table S5 and illustrated in Figures 5E and 5F.

### Tissue-specific ASE genes within the backcross group and their overlap with *cis*-eQTLs

**Figure 6:**
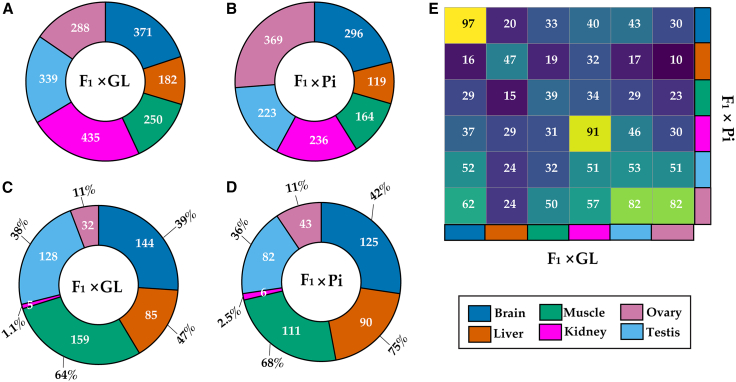
Tissue-specific patterns of allele-specific expression (ASE) and overlap with cis-eQTLs in backcross groups (A and B) The donut plots show the distribution of significant ASE genes across six tissues in backcross groups (A) F_1_ × GL and (B) F_1_ × Pi. (C and D) Proportion of ASE genes overlapping *cis*-eQTLs in backcross groups (C) F_1_ × GL and (D) F_1_ × Pi. (E) The block plot of shared ASE gene counts between F_1_ × GL and F_1_ × Pi tissues. The *x* axis represents tissues from F_1_ × GL, and the *y* axis represents tissues from F_1_ × Pi. Tissue colors: liver (orange), brain (blue), muscle (green), kidney (pink), ovary (magenta), and testis (light blue).

Our one-condition gene-level ASE analysis (*p* < 0.05) across six tissues (brain, liver, muscle, kidney, testis, and ovary) revealed different numbers of ASE genes in both backcross groups, with distinct, tissue-specific patterns that differed between the two genetic backgrounds. In the F_1_ × GL backcross, the kidney exhibited the highest number of ASE genes (435), followed by the brain (371), testis (339), ovary (288), muscle (250), and liver (182; Figure 6A). In contrast, the F_1_ × Pi backcross showed ovary as the tissue with the highest ASE count (369), followed by brain (296), kidney (236), testis (223), muscle (164), and liver (119; Figure 6B). Furthermore, to investigate the regulatory basis of ASE, we compared the identified genes with *cis*-eQTLs from PigGTEx (FDR <0.05). Muscle and liver exhibited the highest overlap in both backcrosses (muscle: F_1_ × GL 64%, F_1_ × Pi 68%; liver: F_1_ × GL 47%, F_1_ × Pi 75%), whereas brain (F_1_ × GL 39%, 144/371; F_1_ × Pi 42%, 125/296) and testis (F_1_ × GL 38%, 128/339; F_1_ × Pi 37%, 82/223) showed moderate concordance. Notably, tissues with the highest ASE gene counts, the kidney in F_1_ × GL and the ovary in F_1_ × Pi, displayed minimal overlap with *cis*-eQTLs (kidney: 1.1–2.5%; ovary: 11%), as shown in Figures 6C and 6D and Table S6. Furthermore, we compared ASE gene sets between F_1_ × GL and F_1_ × Pi across tissues. By comparing gene names, we found the largest same-tissue overlaps in brain (97 shared genes), kidney (91), and ovary (82), with smaller overlaps in liver (47) and muscle (39), indicating that a substantial portion remains tissue- and backcross-specific (Figure 6E).

### Tissue-specific functional enrichment of ASE genes identified within the backcross group

**Figure 7:**
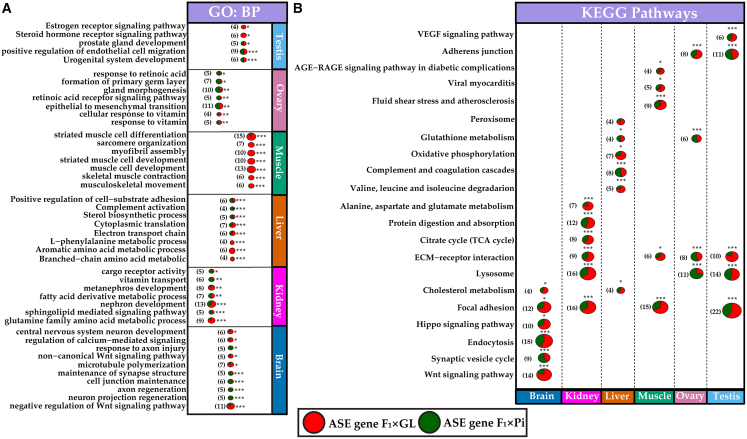
Tissue- and backcross-specific functional enrichment of ASE genes (A and B) Pie chart-style dot plot illustrating function enrichment (A) gene ontology (biological process) and (B) KEGG pathway enrichment. The size of each pie corresponds to the total number of genes in the enriched term (gene count shown in brackets), and pie color indicates the proportion of ASE genes from F_1_ × GL (red) and ASE genes from F_1_ × Pi (dark green). Asterisks represent statistical significance (∗*p* < 0.05, ∗∗*p* < 0.01, ∗∗∗*p* < 0.001).

Functional enrichment analysis on the sets of ASE genes identified in each of the six fetal tissues from the two backcrosses (F_1_ × GL and F_1_ × Pi) revealed strong tissue-specific patterns (Table S7). In the brain, ASE genes from the F_1_ × GL backcross were enriched in neuronal structure and signaling, including microtubule polymerization, non-canonical Wnt signaling, and negative regulation of Wnt signaling, whereas F_1_ × Pi ASE genes were enriched in processes related to neuronal repair and maintenance, such as axon regeneration, response to axon injury, and the maintenance of synapse and cell junction structures. Similarly, KEGG pathway analysis reflected these trends: F_1_ × GL ASE genes were enriched in Wnt signaling, synaptic vesicle cycling, and Hippo signaling, whereas F_1_ × Pi ASE genes were associated with endocytosis and other pathways focused on neuronal repair (Figures 7A and 7B). In the liver, ASE genes from the F_1_ × GL backcross were predominantly associated with metabolic activities, including the breakdown of branched-chain and aromatic amino acids, L-phenylalanine processing, electron transport chain function, cytoplasmic protein synthesis, and sterol production. By contrast, F_1_ × Pi ASE genes were enriched in immune and adhesion-related processes, such as complement activation, regulation of cell-substrate adhesion, and complement and coagulation cascades. KEGG pathway analysis similarly indicated that F_1_ × GL ASE genes were enriched in metabolic pathways, including valine, leucine, and isoleucine degradation, peroxisome, oxidative phosphorylation, glutathione metabolism, and cholesterol metabolism, while F_1_ × Pi ASE genes were enriched in complement and coagulation cascades (Figures 7A and 7B).

Furthermore, in muscle, where nearly all enriched terms for muscle structure and development and contraction, including musculoskeletal movement, skeletal muscle contraction, muscle cell development, striated muscle cell differentiation, myofibril assembly, and sarcomere organization, were exclusively enriched by ASE genes from the F_1_ × GL backcross (Figure 7A). The KEGG pathway analysis indicated that F_1_ × GL ASE genes were enriched in pathways related to muscle function, including focal adhesion and ECM-receptor interaction, whereas F_1_ × Pi ASE genes showed a smaller contribution across these pathways (Figure 7B). Kidney showed a functional distinction between the backcross groups. ASE genes from the F_1_ × GL backcross were enriched for developmental processes, including nephron development and metanephros development. By contrast, F_1_ × Pi ASE genes contributed more to transport and signaling processes, such as vitamin transport, cargo receptor activity, sphingolipid-mediated signaling, and fatty acid derivative metabolism. KEGG pathway revealed that mostly the F_1_ × GL ASE genes were enriched in kidney-relevant pathways, including the citrate cycle (TCA cycle), focal adhesion, and ECM-receptor interactions (Figures 7A and 7B).

Ovary and testis enrichment highlighted processes in cell signaling and adhesion. In the ovary, F_1_ × GL ASE genes were enriched in vitamin-related signaling, including retinoic acid receptor activity and cellular responses to vitamins, whereas F_1_ × Pi ASE genes were associated with developmental and morphogenetic processes, such as primary germ layer formation and gland morphogenesis. KEGG analysis showed F_1_ × GL genes in ECM-receptor interaction and adherens junction pathways, while F_1_ × Pi genes contributed more to lysosome and glutathione metabolism. Similarly, in the testis, F_1_ × GL ASE genes were predominantly associated with steroid hormone signaling and reproductive regulation, including intracellular steroid receptor and estrogen receptor pathways. In contrast, F_1_ × Pi ASE genes were enriched in urogenital system development, endothelial cell migration, and prostate gland formation. Corresponding KEGG analysis indicated enrichment of F_1_ × GL genes in ECM-receptor interaction, focal adhesion, adherens junction, and VEGF signaling, whereas F_1_ × Pi genes showed a smaller contribution, primarily in lysosome-associated pathways (Figures 7A and 7B). The complete enrichment results, including statistical values and the precise proportion of ASE genes from F_1_ × GL and/or F_1_ × Pi in each tissue, are provided in Table S7.

### Tissue-specific differential allele-specific expression between the backcross groups

**Figure 8:**
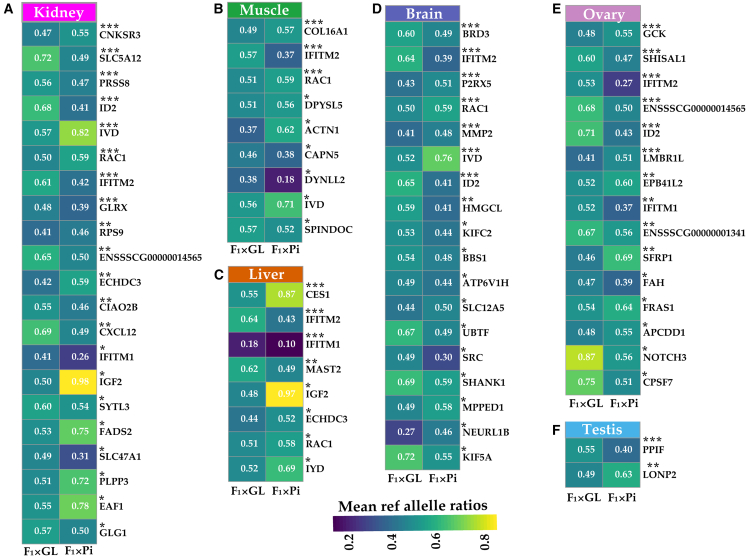
Differential allele-specific expression between backcross groups across six tissues The heatmap depicts genes with significant differences in mean reference allele ratios between the two backcross groups within each tissue. (A–F) Kidney, (B) muscle, (C) liver, (D) brain, (E) ovary, and (F) testis. Asterisks denote statistical significance (∗*p* < 0.05, ∗∗*p* < 0.01, ∗∗∗*p* < 0.001). The color gradient indicates the magnitude and direction of mean reference allele ratios differences between backcross groups.

A beta-binomial GLM was implemented to correct for overdispersion in allelic count data and identify genes showing differential ASE (dASE) between F_1_ × GL and F_1_ × Pi backcross groups across six tissues. Within each tissue, the model tested whether the mean reference allele ratio differed significantly between the two groups. The largest number of significant dASE genes was observed in the kidney (21) and brain (18), followed by the ovary (15), muscle (9), and liver (8), whereas the testis showed minimal differences (2) (Figures 8A–8F). Patterns of allelic ratio differences varied across tissues. In the brain and ovary, most dASE genes had higher mean reference allele ratios in F_1_ × GL (11/18 and 9/15, respectively). In contrast, the liver and muscle displayed a more balanced distribution between groups (liver: 3 F_1_ × GL vs. 5 F_1_ × Pi; muscle: 4 F_1_ × GL vs. 5 F_1_ × Pi). In the kidney, 12/21 genes have higher ratios in F_1_ × GL and 9/21 in F_1_ × Pi. A complete summary of dASE counts and allelic ratio differences per tissue is provided in Table S8. Notably, we identified three genes that consistently differed in mean reference allele ratios across multiple tissues. *IFITM2* had higher mean ratios in F_1_ × GL across five tissues (brain, kidney, liver, muscle, and ovary). *ID2* showed a similar F_1_ × GL bias, but across three tissues (brain, kidney, and ovary). In contrast, *RAC1* consistently showed higher ratios in F_1_ × Pi across the brain, kidney, and liver.

The most prominent dASE genes, showing the largest differences in mean reference-allele ratios (F_1_ × GL vs. F_1_ × Pi), were identified within each tissue. In the kidney, these included *SLC5A12* (0.72 vs. 0.49; *p*adj = 8.79 × 10^-7^), *ID2* (0.68 vs. 0.41; *p*adj = 1.22 × 10^-5^), and *IVD* (0.57 vs. 0.82; *p*adj = 2.20 × 10^-5^). In the brain, the top genes were *BRD3* (0.60 vs. 0.49; *p*adj = 3.11 × 10^-16^) and *IFITM2* (0.64 vs. 0.39; *p*adj = 6.34 × 10^-7^) (Figure 8D). In the ovary, the strongest dASE genes were *SHISAL1* (0.60 vs. 0.47; *p*adj = 5.58 × 10^-13^), *IFITM2* (0.53 vs. 0.27; *p*adj = 5.97 × 10^-12^), and *ID2* (0.71 vs. 0.43; *p*adj = 5.78 × 10^-6^). In the liver, *CES1* (0.55 vs. 0.87; *p*adj = 1.67 × 10^-17^) and *IFITM2* (0.64 vs. 0.43; *p*adj = 7.32 × 10^-5^) were most prominent, whereas in muscle, the largest differences were observed for *COL16A1* (0.49 vs. 0.57; *p*adj = 1.04 × 10^-9^) and *IFITM2* (0.57 vs. 0.37; *p*adj = 7.12 × 10^-7^) as shown in Figures 8A–8E and Table 1.

**Table 1:** Summary of key dASE genes between F_1_×GL and F_1_×Pi backcrosses

Gene	Tissue	DE	dASE directions	eQTL	QTL
*SLC5A12*	Kidney	NS	GL↑ (0.72→0.49)	No	Growth, Carcass
*IVD*	Kidney	Pi↑	Pi↑ (0.57→0.82)	No	Growth, Metab.
*BRD3*	Brain	NS	GL↑ (0.60→0.49)	Yes	Feed eff., Repro.
*CES1*	Liver	NS	Pi↑ (0.55→0.87)	No	Growth, Immune
*ACTN1*	Muscle	NS	Pi↑ (0.48→0.56)	Yes	Meat qual., Carcass
*COL16A1*	Muscle	NS	Pi↑ (0.49→0.57)	Yes	Meat qual., Growth
*ID2*	Brain/kidney/ovary	NS	GL↑ (3 tissues)	Yes	Growth, Repro.
*IFITM2*	Brain/kidney/ovary/liver/muscle	NS	GL↑ (5 tissues)	No	Growth, Immune

NS = not significant (FDR≥0.05); DE = differential expression; Pi↑ = upregulated in F_1_×Pi; GL↑ = reference allele ratio higher in F_1_×GL than in F_1_×Pi; Pi↑ = reference allele ratio higher in F_1_×Pi than in F_1_×GL; → shows mean reference allele ratio (F_1_×GL → F_1_×Pi). eQTL: Yes = *cis*-eQTL support (PigGTEx, FDR<0.05), No = no *cis*-eQTL support; Metab. = metabolism; Feed eff. = feed efficiency; Repro. = reproduction; Meat qual. = meat quality.

### Genomic overlap of ASE genes with porcine QTL intervals

**Figure 9:**
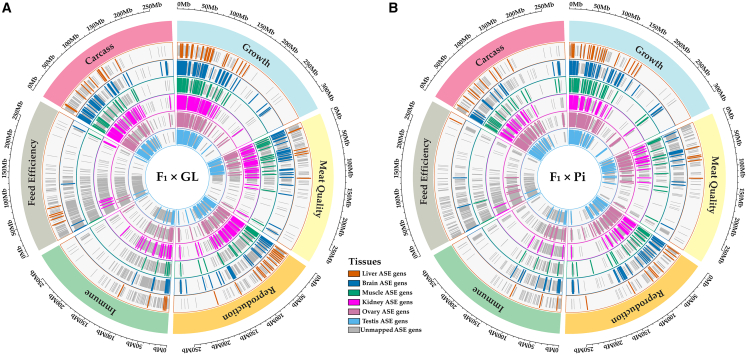
Genome-wide tissue-specific genomic overlap of ASE genes with porcine QTL Circos plots illustrate the overlap between allele-specific expression (ASE) genes and known porcine QTLs in (A) F_1_ × GL and (B) F_1_ × Pi backcrosses. The outer layer represents the genomic positions of QTLs, while the second layer displays six QTL trait categories: growth, reproduction, carcass, meat quality, immune, and feed efficiency. The six inner sectors correspond to tissues: liver (orange), brain (blue), muscle (green), kidney (pink), ovary (magenta), and testis (light blue). Within each sector, lines represent ASE genes: colored lines indicate genes mapped to QTLs in that tissue, whereas gray lines show ASE genes not linked to any QTL.

We assessed the overlap between ASE genes identified across six tissues (brain, kidney, liver, muscle, ovary, and testis) and two backcross groups (F_1_ × GL and F_1_ × Pi) with known porcine quantitative trait loci (QTLs). Because pig QTL intervals can be large in some studies, we first examined the size distribution of our QTLs. The median interval was only 11 bp (Table S9), indicating that our QTLs originate from fine-mapping or SNP-level associations. Growth-related QTLs overlap with the highest number of ASE genes across all tissues. In F_1_ × GL, the largest overlaps were observed in ovary (80/288 genes, 27.8%), kidney (95/435 genes, 21.8%), testis (72/339 genes, 21.2%), brain (78/371 genes, 21.0%), muscle (50/250 genes, 20.0%), and liver (31/182 genes, 17.0%), whereas in F_1_ × Pi, the highest overlaps were in ovary (88/369 genes, 23.8%) and muscle (39/164 genes, 23.8%), followed by kidney (49/236 genes, 20.8%), testis (45/223 genes, 20.2%), brain (54/296 genes, 18.2%), and liver (21/119 genes, 17.6%). Reproduction-related QTLs overlapped with the largest numbers of ASE genes in F_1_ × GL testis (46/339 genes, 13.6%) and ovary (37/288 genes, 12.8%), and in F_1_ × Pi ovary (47/369 genes, 12.7%) and muscle (18/164 genes, 11.0%). Carcass-related QTLs overlap with the highest numbers of ASE genes in F_1_ × GL kidney (57/435 genes, 13.1%) and brain (45/371 genes, 12.1%), and in F_1_ × Pi testis (32/223 genes, 14.3%) and kidney (24/236 genes, 10.2%). Meat Quality-related QTLs overlapped with the largest numbers of ASE genes in F_1_ × GL brain (37/371 genes, 10.0%) and kidney (39/435 genes, 9.0%), and in F_1_ × Pi muscle (13/164 genes, 7.9%) and ovary (25/369 genes, 6.8%). Immune-related QTLs overlap with the highest numbers of ASE genes in F_1_ × GL ovary (15/288 genes, 5.2%) and kidney (19/435 genes, 4.4%), and in F_1_ × Pi brain (11/296 genes, 3.7%) and testis (8/223 genes, 3.6%). Feed Efficiency-related QTLs consistently contained few ASE genes, with the highest numbers in F_1_ × GL brain (6/371 genes, 1.6%) and kidney (6/435 genes, 1.4%), and in F_1_ × Pi ovary (7/369 genes, 1.9%) and testis (3/223 genes, 1.3%), as shown in Figures 9A and 9B. To assess whether observed overlaps exceed random expectation, we performed a permutation test (10,000 randomizations). For all tissue-backcross combinations, the observed number of ASE genes overlapping QTL intervals was significantly greater than expected by chance (empirical *p* < 0.05; Table S9). The complete ASE gene-QTL category mapping across tissues is provided in Table S10.

## Discussion

Our multi-tissue analysis reveals that paternal genetic background is a key driver of fetal transcriptional programming, shaping development through a core trade-off between metabolic capacity and developmental plasticity. Differential ASE was widespread across tissues, with the kidney and brain showing the strongest sire-specific divergence. Crucially, our study moves beyond correlative expression changes by applying a haplotype-agnostic framework to detect ASE and to pinpoint putative *cis*-regulatory variants via dASE, thereby linking sire genetics to breed-specific developmental phenotypes. The genomic overlap of these regulatory effects with known QTLs for growth, metabolism, and reproduction provides a direct link between paternal-derived fetal programming and the genetic architecture of complex production traits.

Global transcriptomic divergence revealed that F_1_ × GL conceptuses at GD63 prioritize metabolic and biosynthetic processes, including amino acid metabolism, oxidative phosphorylation, and purine metabolism. This aligns with studies showing that maternal nutritional stressors converge on mitochondrial energy production pathways,^36^^,^^37^^,^^38^ positioning paternal genetics as an equally key contributor to developmental metabolic programming. In contrast, F_1_ × Pi conceptuses upregulated a suite of developmental and regulatory processes, oogenesis, neural crest differentiation, and cell cycle control, suggesting the Pi paternal lineage actively biases offspring toward rapid growth and developmental plasticity through targeted transcriptional modulation, a process often governed by epigenetic mechanisms.^39^^,^^40^^,^^41^ Sire breed effects were tissue-specific at gestational day 63, a critical stage for organ formation and differentiation.^42^^,^^43^^,^^44^ Across tissues, GL sires favored metabolic robustness and structural maturation, whereas Pi sires promoted stress responsiveness and developmental plasticity. Fetal growth in pigs is largely determined by maternal and uterine-placental factors (e.g., uterine capacity, placental efficiency, and maternal nutrient/endocrine supply), which can drive adaptive changes in the fetal transcriptome and epigenome.^45^^,^^46^^,^^47^ Consistent with this, piglet birth weight is reported to be influenced predominantly by maternal genetic effects.^48^ We previously identified GD63 fetal-muscle transcripts associated with extreme fetal weights^49^ and the correlation of transcript levels with fetal weight.^50^ In the present study, fetuses were not selected for extreme weights; thus, sire-associated transcriptional differences likely reflect paternal programming of tissue-specific developmental trajectories, while gross fetal weight remains buffered by maternal-placental homeostasis at GD63.

In the fetal brain, where GD63 corresponds to active neurite outgrowth and synaptogenesis in pigs,^51^^,^^52^ GL-sired fetuses favored synaptogenic pathways, including synapse organization, regulation of synaptic activity, and chemical transmission. Functional enrichment analysis of the ASE gene from the F_1_ × GL backcross further highlighted Wnt signaling and microtubule polymerization, canonical drivers of neurite outgrowth and circuit assembly.^53^^,^^54^ In contrast, Pi-sired fetuses showed ASE enrichment for axon regeneration, synapse maintenance, and endocytosis, consistent with protective and repair-oriented neural programs.^55^^,^^56^ Notably, GL-biased synaptogenesis co-occurred with ferroptosis-related gene enrichment, suggesting a trade-off between rapid neural maturation and increased oxidative vulnerability.^57^^,^^58^ This tissue-specific pattern of ASE is consistent with the genetic architecture of production traits: a substantial proportion of brain ASE genes overlap with growth (F_1_ × GL: 21.0%; F_1_ × Pi: 18.2%) and meat quality QTLs (F_1_ × GL: 10.0%), suggesting that breed-associated ASE difference on early neurodevelopment may align with loci that influence feed efficiency, growth, and meat characteristics.

In F_1_ × GL fetuses at gestational day 63, the liver favored hepatocyte differentiation and mitochondrial activity, consistent with enrichment of NAD^+^ metabolism, PPAR pathways, and retinoid processing. This aligns with a study reported that PPARα-RXRA signaling enhances fatty acid oxidation and retinoid metabolism in sheep,^59^ and that elevated hepatic NAD^+^ promotes mitochondrial oxidation and metabolic flexibility in rodents.^60^ ASE genes enrichment for amino acid degradation, oxidative phosphorylation, and peroxisomal activity indicates a program tuned for efficient ATP generation, reinforcing a high-energy state that supports accelerated growth and may shape postnatal metabolic function.^61^^,^^62^^,^^63^ By contrast, F_1_ × Pi fetal livers upregulated adipocytokine signaling, a pathway known to regulate lipid handling and insulin sensitivity *in utero*,^64^ and ASE genes were enriched for immune processes, including complement activation, consistent with studies showing early complement expression is essential for shaping hepatic immune function.^65^ Thus, GL sires bias the fetal liver toward anabolic and energy-intensive metabolism, whereas Pi biases toward metabolic adaptability and immune regulation, a divergence that may underlie differences in postnatal metabolic resilience. This functional divergence is reflected at the genomic level: 17.0% of F_1_ × GL and 17.6% of F_1_ × Pi liver ASE genes overlap Growth QTLs, suggesting that breed-associated ASE difference on prenatal liver metabolism may contribute to the genetic potential for postnatal growth efficiency.

In fetal muscle, sire breed effects primarily reflect differences in the timing of gene expression rather than the number of genes expressed. At GD63, GL-sired fetuses showed early enrichment of pathways involved in sarcomere organization, myofibril assembly, and contraction, coinciding with the critical window of secondary myofiber formation.^66^ Comparative transcriptomic profiling across breeds revealed muscle-specific gene expression shifts associated with postnatal muscularity.^67^ Notably, genes exhibiting ASE in F_1_ × GL fetuses were enriched for similar structural pathways, suggesting accelerated prenatal maturation of myofiber architecture. These findings align with previous studies showing that prenatal myogenesis determines fiber number and early structural organization, which influence postnatal growth and meat quality.^68^ Although Piétrain pigs are renowned for extreme muscularity, their fetuses rely more on postnatal hypertrophy, whereas GL fetuses initiate structural maturation earlier during secondary myogenesis.^67^^,^^69^ This developmental strategy is reflected in the genetic architecture of production traits: 20.0% of F_1_ × GL and 23.8% of F_1_ × Pi ASE genes in fetal muscle overlap growth QTLs, with up to 8.0% overlapping meat quality and 10.4% (Pi) overlapping carcass QTLs, highlighting a direct link between breed-associated ASE difference on prenatal myogenesis and the genomic control of muscle mass and meat attributes.

The kidney exhibited the most pronounced sire-breed divergence in transcriptional profiles at GD63. In F_1_ × GL fetuses, genes associated with acyl-CoA metabolism, ER protein processing, and TCA cycle activity were upregulated, suggesting that F_1_ × GL contributes mainly to metabolic and processing functions to support nephron differentiation. This aligns with prior evidence that acetyl-CoA metabolism is indispensable for nephron progenitor maintenance and nephrogenesis, as perturbation of ATP-citrate lyase disrupts nephron formation and reduces glomerular endowment.^70^ Nephron progenitors shift from glycolysis during proliferation to fatty acid oxidation and oxidative phosphorylation upon differentiation,^71^ while proximal tubules rely heavily on fatty acid oxidation and the TCA cycle to sustain ATP demands.^72^ ER protein processing upregulation also reflects the essential role of the unfolded protein response in renal proteostasis and lipid metabolism.^73^ In contrast, F_1_ × Pi emphasizes stress response, ion transport, and developmental pathways central to kidney physiology. Previous studies have shown that oxidative stress responses are critical determinants of nephron progenitor cell fate and developmental plasticity.^74^ Transcriptional regulators such as Tfap2a also align nephron terminal differentiation with solute transporter gene expression.^75^ Our findings extend these observations: F_1_ × GL ASE genes were enriched for nephron and metanephros development, as well as kidney-relevant pathways, including the citrate cycle, focal adhesion, and ECM-receptor interactions, highlighting alleles that promote energy metabolism and protein homeostasis.^71^ In contrast, F_1_ × Pi ASE genes were enriched for oxidative stress responses, transport, and signaling pathways, suggesting Pi sires promote adaptive kidney development. These sire-specific ASE patterns suggest distinct effects on renal metabolism, developmental trajectory, and long-term functional capacity. A substantial proportion of kidney ASE genes, 21.8% in F_1_ × GL and 20.8% in F_1_ × Pi, overlap with Growth QTLs, with further overlap with carcass (up to 13.1%) and reproduction (up to 12.9%) QTLs, indicating that the metabolic and developmental programs in the fetal kidney are embedded within the genomic regions that shape growth efficiency, body composition, and reproductive potential. In the ovary and testis, ASE profiles diverged in parallel to modulate gonadal development. F_1_ × GL ASE genes were enriched for vitamin signaling and steroid receptor activity, whereas F_1_ × Pi ASE genes were enriched for morphogenetic and migration pathways, indicating that *cis*-regulatory variation shapes mid-gestation gonadal programs. Consistent with their biological function, the specific enrichment of ASE genes in gonadal tissues within reproduction QTLs (testis, F_1_ × GL: 13.6%; ovary, F_1_ × Pi: 12.7%), coupled with strong overlap with Growth QTLs (ovary, F_1_ × GL: 27.8%; testis, F_1_ × GL: 21.2%), suggests that paternal genetic effects on fetal reproductive development may link to the genetic control of both reproductive efficiency and overall growth potential.

Importantly, our dASE analysis identified key candidate genes where allelic imbalance provides a direct mechanistic link to the observed transcriptomic phenotypes. In the kidney, GL-derived allele bias at *SLC5A12* (solute carrier family 5 member 12, a monocarboxylate transporter) may enhance substrate uptake for the TCA cycle, linking *cis*-regulatory variation to GL-associated metabolic programs.^76^ Conversely, a Pi-derived allele bias at *IVD* (isovaleryl-CoA dehydrogenase), which catalyzes a key step in leucine catabolism, may enhance branched-chain amino acid metabolism and TCA-cycle substrate supply, reflecting Pi-associated energy programs.^77^ In the fetal brain, the higher GL-derived allele ratio at *BRD3* (bromodomain containing 3) suggests GL-biased upregulation of synaptogenic programs. This may promote a pro-transcriptional chromatin state at neurodevelopmental loci, aligning with the BET protein roles in neuronal transcription and potentially contributing to accelerated neural maturation.^78^^,^^79^ Notably, *BRD3* resides within QTL intervals for feed efficiency and reproduction, linking this breed-associated ASE difference on brain development to the genetic architecture of these production traits. In fetal muscle, Pi-derived allele biases at *ACTN1* (actinin alpha 1) and *COL16A1* (collagen type XVI alpha 1 chain) provide a *cis*-regulatory basis for Pi-biased enrichment of contractile and extracellular matrix pathways. *ACTN1* is essential for sarcomere integrity and Z-disk assembly, while *COL16A1* organizes the extracellular matrix and facilitates myofibril adhesion, suggesting that these biases may contribute to accelerated muscle maturation in Pi-sired fetuses.^80^^,^^81^ These key structural genes overlap with QTLs for meat quality, carcass, and growth, directly connecting their allele-specific expression to the genetic control of core muscularity traits. In the liver, Pi-derived allele bias at CES1 (carboxylesterase 1) is associated with Pi-biased upregulation of xenobiotic metabolism, supporting detoxification pathways.^82^ Additionally, Pi-biased expression at *IGF2* (insulin-like growth factor 2) suggests a coordinated mechanism in which *IGF2* promotes anabolic growth while CES1 enhances metabolic processing.^83^ Replicable allele-specific effects at *ID2* (inhibitor of DNA binding 2), *IFITM2* (interferon-induced transmembrane protein 2), and *RAC1* (Rac family small GTPase 1) suggest these genes may act as master regulators, spreading *cis*-regulatory differences across multiple organs and coordinating breed-specific developmental programs.

Paternal breed exerts a profound and tissue-specific influence on fetal programming, shaping transcriptional networks and allele-specific expression patterns at gestational day 63. Our results demonstrate that GL genetics promotes metabolic capacity and accelerated maturation, while Pi genetics enhances stress responsiveness and developmental plasticity. The concordance between differential expression and differential allele-specific expression patterns identifies key candidate genes, including nutrient transporters *(SLC5A12)*, metabolic enzymes *(IVD* and *CES1)*, chromatin regulators *(BRD3)*, and structural components *(ACTN1* and *COL16A1)*, through which *cis*-regulatory divergence may drive breed-specific developmental programs. These results establish paternal genetics as a critical factor shaping prenatal development, with clear implications for postnatal metabolism, growth, and organ function. The prioritized candidate genes provide a foundation for functional validation and for translating paternal genomic effects into breeding strategies and mechanistic insights into fetal programming, directly linking sire-derived *cis*-regulatory variation to the genetic control of commercially important traits.

### Limitations of the study

While our dASE analysis robustly identifies loci influenced by paternal genetics, this study has several key limitations. First, the haplotype-agnostic approach, while advantageous for comprehensive discovery, limits our ability to definitively distinguish whether the observed allelic imbalances are driven by *cis*-regulatory variation or unrecognized genomic imprinting. Although the high overlap between our ASE genes and *cis*-eQTLs from the PigGTEx resource suggests a predominant *cis*-regulatory basis for most signals, this remains an inference for individual loci. In addition, because parent-of-origin information was unavailable for ASE analysis, these signals were interpreted as breed-associated allele-specific expression rather than paternal or maternal effects. Future studies incorporating phased genotypes or parental information will be needed to resolve parent-of-origin effects. Second, our data reflect a single, static observation at gestational day 63, capturing a critical developmental window but missing the dynamic progression of allelic expression patterns throughout gestation. This snapshot cannot account for the inherent variability and individuality of ontogenetic development. Post-hoc power analysis under the observed sample sizes showed limited sensitivity for small-effect variants. Therefore, the low number of dASE genes observed in gonadal tissues, especially the testis, likely reflects reduced power in addition to possible biological differences. Furthermore, in our analyses, sex did not appear to exert a major influence on the transcriptomic profiles, as sex was included as a fixed effect in the models. However, because we did not formally test sex-by-backcross interactions, we cannot rule out subtle sex-specific effects that may emerge in larger cohorts. In addition, although QTL overlap analysis showed significant enrichment after permutation testing, QTL intervals were defined only by genomic coordinates. Formal colocalization will require Bayesian fine-mapping or functional validation. Finally, our findings are based on statistical inference and correlation; functional validation through approaches like pyrosequencing or CRISPR-based perturbations is required to establish a direct mechanistic link between the identified candidate genes and the observed developmental phenotypes. Future studies incorporating phased parental genomes and longitudinal designs are needed to resolve these mechanisms and determine the functional relevance of these fetal molecular events for postnatal outcomes.

## Resource availability

### Lead contact

Further information and requests for resources should be directed to and will be fulfilled by the lead contact, Siriluck Ponsuksili (ponsuksili@fbn-dummerstorf.de).

### Materials availability

This study did not generate new, unique reagents.

### Data and code availability

• Raw RNA-seq data have been deposited at the EMBL-EBI ArrayExpress: https://www.ebi.ac.uk/arrayexpress/experiments/E-MTAB-15588. Pig QTL data were obtained from the Animal QTL Database (QTLdb; Release 58, December 29, 2025): https://www.animalgenome.org/cgi-bin/QTLdb/SS/index. The *Sus scrofa* reference genome Sscrofa11.1 and annotations were obtained from ENSEMBL Release 113.
• All custom R scripts for statistical analyses and visualization are available at the GitHub repository: https://github.com/MuhammadArsalanIqbal/Fetal_Programming_ASE_Analysis. The repository includes three R Markdown workflows: (i) differential expression analysis using DESeq2 with Surrogate Variable Analysis (SVA), (ii) allele-specific expression detection using the ASEP package, and (iii) differential ASE analysis using a beta-binomial generalized linear model with the VGAM package, along with all figure generation scripts.
• Any additional information required to reanalyze the data reported in this study is available from the lead contact upon reasonable request.

## Acknowledgments

The authors thank Nicole Gentz, Annette Jugert, and Joana Bittner for excellent technical assistance.

## Author contributions

Conceptualization, E.M., K.W., and S.P.; data curation, E.M. and N.T.; formal analysis, M.A.I. and F.H.; investigation, M.O. and H.R.; methodology, F.H. and N.T.; software, M.A.I. and F.H.; supervision, S.P.; writing – original draft, M.A.I.; writing – review and editing, E.M., M.O., H.R., N.T., K.W., and S.P. All authors reviewed and approved the manuscript.

## Declaration of interests

The authors declare no competing interests.

## STAR★Methods

### Key resources table

**Table:** 

REAGENT or RESOURCE	SOURCE	IDENTIFIER
**Biological samples**		

F_1_×GL and F_1_×Pi fetal tissues (liver, brain, muscle, kidney, ovary, testis)	This study	N/A

**Chemicals, peptides, and recombinant proteins**		

QIAzol Lysis Reagent	Qiagen	Cat#79306
DNase	Qiagen	Cat#79254
RNeasy Mini Kit	Qiagen	Cat#74106
Illumina Stranded mRNA Prep Ligation kit	Illumina	Cat#20040534
NextSeq 2000 P3 Reagents kit (200 cycles)	Illumina	Cat#20040560
Agilent RNA 6000 Nano Kit	Agilent Technologies	Cat#5067-1511

**Deposited data**		

Raw RNA-seq reads	This study	ArrayExpress: E-MTAB-15588
Custom R scripts (Fetal Programming ASE Analysis)	This study	GitHub: https://github.com/MuhammadArsalanIqbal/Fetal_Programming_ASE_Analysis

**Software and algorithms**		

FastQC v0.11.9	Babraham Institute	https://www.bioinformatics.babraham.ac.uk/projects/fastqc/
Trim Galore v0.6.7	Krueger^84^	https://github.com/FelixKrueger/TrimGalore
HISAT2 v2.2.1	Kim et al.^85^	https://daehwankimlab.github.io/hisat2/
HTSeq v0.11.1	Putri et al.^86^	https://htseq.readthedocs.io/
STAR v2.7.8a	Dobin et al.^87^	https://github.com/alexdobin/STAR
GATK v4.2.0.0	Broad Institute	https://gatk.broadinstitute.org/
bedtools v2.27.1	Quinlan & Hall^88^	https://bedtools.readthedocs.io/
SAMtools v1.12	Li et al.^89^	http://www.htslib.org/
ASEP v0.1.0	Fan et al.^35^	https://jiaxin-fan.github.io/ASEP/articles/introduction.html
DESeq2 v1.44.0	Love et al.^90^	https://bioconductor.org/packages/DESeq2/
sva v3.52.0	Leek et al.^91^	https://bioconductor.org/packages/sva/
VGAM	Yee et al.^92^	https://cran.r-project.org/package=VGAM
vegan v2.7.1	Dixon^93^	https://cran.r-project.org/package=vegan
GenomicRanges v1.52.1	Lawrence et al.^94^	https://bioconductor.org/packages/GenomicRanges/
circlize v0.4.15	Gu et al.^95^	https://cran.r-project.org/package=circlize
pheatmap v1.0.12	Kolde	https://cran.r-project.org/package=pheatmap
ClueGO v2.5.10	Bindea et al.^96^	https://apps.cytoscape.org/apps/cluego
CluePedia v1.5.10	Bindea et al.^97^	https://apps.cytoscape.org/apps/cluepedia
Cytoscape v3.10.2	Shannon et al.^98^	https://cytoscape.org/
ggplot2 v3.5.1	Wickham^99^	https://ggplot2.tidyverse.org/
R (version 4.3.0)	R Core Team	https://www.r-project.org/

**Other**		

Precellys 5000 homogenizer	Bertin Instruments	N/A
NanoDrop ND-2000 spectrophotometer	ThermoFisher	N/A
Bioanalyzer 2100	Agilent Technologies	N/A
NextSeq 2000 system	Illumina	N/A
Pig QTL data	Animal QTLdb	Release 58 (Dec 2025); https://www.animalgenome.org/QTLdb
Sus scrofa reference genome Sscrofa11.1	ENSEMBL	Release 113
dbSNP for Sus scrofa	Ensembl	v94

### Experimental model and study participant details

#### Animals and ethics approval

Animal care and tissue collection procedures were conducted in accordance with established guidelines to ensure good scientific practice and animal welfare and complied with the European Communities Council Directive of 24 November 1986 (86/609/EEC) and the German Animal Welfare Act regulations applicable at the time of sample collection (2009–2010). The fetuses used in this study originated from pregnant sows that underwent routine slaughter as part of standard husbandry and meat production practices. Slaughter was performed using electrical stunning followed by exsanguination, in accordance with best-practice slaughter procedures and the animal welfare regulations applicable at the time. The sows were not subjected to any experimental treatment, diagnostic sampling, or other study-related interventions prior to slaughter. No additional procedures were performed specifically for the purposes of this study. Fetal tissues were collected postmortem, after completion of the slaughter process and consequent death of the fetuses. Therefore, under the regulations in force at that time, specific ethical approval was not required. All handling and slaughter procedures complied with applicable legal requirements and animal welfare standards.

#### Animal model, genotype, and backcross design

The dataset used in this study was derived from a previous project employing a backcross (BC) design, as detailed in.^26^ Briefly, twenty-two F_1_ sows were generated by crossing German Landrace (GL) dams with Piétrain (Pi) sires. These F_1_ sows were then backcrossed to three GL sires and three Pi sires to produce F_1_ × GL and F_1_ × Pi conceptuses, respectively. All fetuses had identical maternal genetic backgrounds (F_1_) but differed in paternal sire breed (GL vs. Pi). The resulting backcross fetuses were therefore 75% GL (F_1_ × GL) or 75% Pi (F_1_ × Pi) in genetic composition. Fetuses were collected at gestational day 63 (GD63), a critical developmental window corresponding to the mid-gestation period in pigs, during which organogenesis and tissue differentiation are actively ongoing.

#### Age and developmental stage

Fetuses were collected at gestational day 63 (GD63), with gestational age confirmed by breeding records. GD63 in pigs corresponds to a mid-gestation developmental stage, characterized by approximately 70% of skeletal muscle fiber formation, completion of neural tube closure, key neurodevelopmental processes, and initiation of gonadal differentiation.

#### Sex distribution and sample size

A total of 38 fetuses were used in this study. For the four non-gonadal tissues (liver, brain, muscle, kidney), 24 matched sample sets were obtained from the same 24 fetuses, comprising 12 F_1_ × GL (6 females, 6 males) and 12 F_1_ × Pi (6 females, 6 males). For gonadal tissues, samples were collected from 17 females (ovary: 9 F_1_ × GL, 8 F_1_ × Pi) and 18 males (testis: 10 F_1_ × GL, 8 F_1_ × Pi), with 10 ovaries and 11 testes originating from the same 24 fetuses used for non-gonadal tissues. The remaining gonadal samples were from additional fetuses included specifically to increase statistical power for gonad-specific analyses. Sample allocation was based on balanced representation across backcross groups, sex, dams, and sires, with the study design prioritizing the availability of all six target tissues from the same fetuses where possible. Tissues (liver, brain, muscle, kidney, ovary, and testis) were carefully dissected, immediately snap-frozen in liquid nitrogen, and stored at −80°C until RNA extraction.

#### Housing and husbandry

Sows were housed under standard conditions with *ad libitum* access to water and a commercial diet. No experimental treatments were applied before slaughter.

### Method details

#### RNA isolation, library construction, and sequencing

Total RNA was isolated from tissue samples using QIAzol Lysis Reagent (Qiagen, Cat# 79306, Germany), while the ovary, testis, and muscle tissues underwent additional homogenization with ceramic beads using a Precellys 5000 homogenizer. These tissues were chosen for homogenization as they are denser and more fibrous, requiring additional mechanical disruption for effective RNA extraction. Following RNA extraction, DNase (Qiagen, Cat# 79254, Germany) treatment was applied to remove genomic DNA contamination, and RNA was purified using the RNeasy Mini Kit (Qiagen, Cat# 74106, Germany). RNA concentration and quality were assessed using a NanoDrop ND-2000 spectrophotometer (ThermoFisher, Germany) and a Bioanalyzer 2100 (Agilent Technologies, Agilent RNA 6000 Nano Kit, Germany). To prepare the RNA-seq library, 1 μg of total RNA with a RIN >8 was used for library preparation using an Illumina Stranded mRNA Prep, Ligation kit with 11 PCR-cycles of amplification according to the manufacturer’s recommendation (Illumina, Cat#20040534, Germany). The libraries were quality checked for fragment length distribution on Agilent 2100 Bioanalyzer and normalized to an equal concentration of 10 nM before pooling. The adaptor-tagged DNA libraries were sequenced at 750 pM final concentration to generate 101-base-pair paired-end reads on the NextSeq 2000 system using a P3 flowcell (NextSeq 2000 P3 Reagents kit, 200 cycles: 20040560, Illumina, Germany) at the sequencing facility of Research Institute for Farm Animal Biology (FBN), Dummerstorf, Germany.

#### Read preprocessing and alignment

The raw sequencing reads underwent quality control using FastQC (version 0.11.9),^100^ and adapter sequences were trimmed using Trim Galore (version 0.6.7).^84^ Low-quality reads with an average Q-score below 20 were excluded, and adapter-like sequences were filtered out. Afterward, high-quality paired-end reads were mapped to the Sscrofa11.1 reference genome (ENSEMBL release 113) using HISAT2 (version 2.2.1),^85^ achieving a 98% mapping rate. Read quantification was performed using HTSeq (version 0.11.1).^86^ Raw fastq files and metadata are available in the ArrayExpress database under accession number E-MTAB-15588.

#### Transcriptome-derived variant profiling with GATK

To ensure robust variant discovery and accurate genotyping, RNA-seq data were processed following the GATK Best Practices guidelines (v4.2.0.0).^31^^,^^32^ The STAR aligner (version 2.7.8a) was used in two-pass alignment mode to map paired-end reads to the Sus scrofa reference genome (Sscrofa11.1), thereby optimizing splice junction detection.^87^ Post-alignment, quality control steps were applied to retain only high-confidence reads. Subsequently, reads with spliced alignments (‘N' in CIGAR strings) were split using “SplitNCigarReads” to enable proper analysis of splicing patterns. Base quality score recalibration was performed using known variants from Ensembl v94’s dbSNP.^101^ Finally, PCR duplicate reads were identified and removed using “GATK’s MarkDuplicates” to avoid false-positive variant calls.

Variant detection was carried out using GATK HaplotypeCaller, which reconstructs haplotypes through local *de novo* assembly to accurately identify single-nucleotide polymorphisms (SNPs) and insertions/deletions (Indels) in expressed genomic regions. To improve the specificity of variant detection, soft-clipped bases were excluded, and variants were called with a minimum confidence score of 20.^102^ Furthermore, variant filtration was conducted using GATK’s VariantFiltration tool by excluding those with significant strand bias (FS > 30.0), low-quality variants (QD < 2.0), and variant clustering artifacts (defined by a cluster size of ≥3 variants within a 35 bp window).

#### Allele-specific expression quantification and one-condition analysis with ASEP

To perform allele-specific expression (ASE) analysis, we implemented an additional GATK Best Practices iteration to reduce reference mapping bias. A masked reference genome was generated by replacing all biallelic SNP positions (identified in Section 2.5) with “N” using bedtools (version 2.27.1).^88^ This N-masked reference was used for STAR two-pass realignment to minimize alignment bias at known variant sites. To further correct for mapping bias in ASE analyses, Weighted Analysis to account for Selection and Population structure (WASP) filtering was applied during this second iteration, ensuring inclusion of only uniquely remapped reads with samtools (version 1.12).^89^ Following alignment, GATK ASEReadCounter was used to quantify reference and alternate allele depths at each original SNP position using the unmasked VCF. To ensure high-confidence SNP-level ASE calls, we retained only heterozygous sites with ≥50 total reads, ≥5 reads per allele, and ≥1% allelic contribution from each allele. Additionally, SNPs located on sex chromosomes and unmapped contigs were excluded from downstream analysis. The resulting high-confidence heterozygous SNP-level counts were aggregated to generate per-gene reference and alternate allele counts for input into gene-level ASE analysis.

Subsequently, ASE detection was performed independently for each tissue–backcross combination across six tissues (brain, liver, muscle, kidney, testis, and ovary) and two backcross groups (F_1_ × GL and F_1_ × Pi) using the ASEP (Allele-Specific Expression Analysis in a Population, version 0.1.0) package within the R programming environment.^35^ The *‘ASE_detection()’* functions were applied to identify gene-level ASE effects with statistical significance (*p*-value <0.05) within each tissue–backcross group. The analyses were performed using unphased, adaptive configurations with a resampling rate of 1e4, and on average, approximately 60% of individuals contributed to the ASE signal per group. Because parental phase information was unavailable, the results reflect allele-specific expression relative to the reference allele and cannot be assigned to paternal or maternal origin. Following ASE detection, we evaluated the potential regulatory relevance of the identified ASE genes by comparing them with *cis*-eQTLs retrieved from the PigGTEx portal within the FarmGTEx database. In the PigGTEx resource, *cis*-eQTLs are defined as genetic variants (SNPs) located within 1 megabase (Mb) upstream or downstream of a gene’s transcription start site that show a significant association (FDR <0.05) with the expression of that gene, with associations tested independently in each tissue using linear model-based eQTL mapping of genotype dosages against normalized expression levels; detailed methodology is provided in.^103^ ASE genes from the F_1_ × GL and F_1_ × Pi backcrosses across six tissues (brain, liver, muscle, kidney, testis, and ovary) were matched against eQTLs from the corresponding tissues in the PigGTEx dataset, filtered at FDR <0.05. The number and proportion of genes shared between ASE and eQTL datasets were visualized using donut plots generated with the ggplot2 package (version 3.5.1) in R.

### Quantification and statistical analysis

#### Differential expression analysis

Gene expression analysis was performed on RNA-seq data obtained from six tissues (brain, liver, muscle, kidney, ovary, and testis) dissected from 38 backcross fetuses, resulting in a total of 131 sequenced libraries. The initial dataset included raw counts for 35,413 genes. After filtering for sufficient expression (≥5 counts in at least 80% of the samples), the tissue-specific filtering resulted in a final set of retained genes for differential expression analysis: brain (15,188 genes), liver (13,460), muscle (14,009), kidney (15,915), ovary (17,908), and testis (17,195). This filter was applied to minimize technical noise and false positives in differential expression testing, as low-count genes are prone to high variability and can inflate false discovery rates, a standard practice in RNA-seq workflows using count-based models such as DESeq2. Applying the same filter to the full set of 131 libraries retained 14,024 genes for the across-tissue backcross comparison. Variance-stabilizing transformation (VST) was applied to the filtered counts to minimize mean-variance dependence.

To account for latent, unobserved confounders, surrogate variable analysis (SVA) was performed using the sva R package (v3.52.0) for each tissue independently.^91^ SVA was conducted independently for each tissue dataset. The number of significant surrogate variables (SVs) was determined using the (num.sv, method = “BE”) function.^104^ This analysis identified the following number of SVs per tissue: liver (2); brain (3); muscle (1); kidney (3); ovary (2); and testis (2). For the BC group comparison across all tissues, SVA identified 8 surrogate variables. These SVs represent unmodeled sources of variation in the data and were included in subsequent differential expression models to minimize confounding.

Differential expression analysis was performed using the R package DESeq2 (version 1.44.0;^90^) with three different modeling strategies: (1) For tissue-specific backcross effects in non-gonadal tissues (brain, liver, muscle, kidney), the model included backcross group, sex, and tissue-specific surrogate variables. (2) For tissue-specific backcross effects in gonadal tissues (ovary, testis), sex was excluded because these tissues are inherently sex-specific, and the model included backcross group, gonadal tissue, and tissue-specific surrogate variables. (3) To assess backcross effects across all tissues, a model was fitted including backcross group, tissue, sex, and the eight surrogate variables estimated from the combined dataset. The differentially expressed genes (DEGs) were identified using Wald tests and adjusted *p*-values (padj <0.05) based on the Benjamini–Hochberg method to control the false discovery rate. The DEGs were visualized using volcano plots generated with the ggplot2 package (version 3.5.1) within R.^99^

#### Principal component analysis and transcriptomic divergence

Principal component analysis (PCA) was performed on variance-stabilizing transformed (VST) gene expression data to assess global and tissue-specific transcriptomic variation between BC groups. A global PCA was performed on the complete dataset, including all tissues and BC groups, to identify major components of variation. For tissue-specific analyses, PCAs were conducted using the SVA-adjusted differentially expressed genes (DEGs) identified between F_1_ × GL and F_1_ × Pi to evaluate within-tissue group separation and quantify BC-driven divergence. This ensured that the visualized separation between backcross groups reflected biological divergence rather than technical variation. Transcriptomic divergence was further measured by two complementary metrics: (1) the DEG-based divergence score (Δ), defined as the mean squared difference in average expression between BC groups normalized by the number of DEGs; and (2) a normalized separation distance, defined as the Euclidean distance between group centroids in PC1–PC2 space divided by the square root of the number of DEGs √(n_DEGs) to account for differences in DEG set size across tissues, enabling cross-tissue comparison.

#### Differential ASE genes between backcross groups within each tissue

The reference allele is defined based on the Sscrofa11.1 reference genome and is therefore neutral and not inherently biased toward either parental breed. To identify genes with significant differences in allelic imbalance (dASE) between the backcross groups (F_1_ × GL vs. F_1_ × Pi), analyses were performed for each of the six tissues. The analysis was restricted to the union of genes previously identified as exhibiting significant ASE (*p*-value <0.05) in either the F_1_ × GL or F_1_ × Pi group for that specific tissue from our initial one-condition ASEP analysis, ensuring a focused investigation on genes with active allelic regulation. A beta-binomial generalized linear model (GLM) was employed to account for overdispersion in the allele-specific count data, implemented using the vglm() function from the VGAM R package.^92^ To ensure reliable and representative dASE estimation, genes were retained if they had valid allele-specific read counts for both reference and alternate alleles, were supported by three or more heterozygous SNPs, and were non-missing in at least 60% of individuals per group within each tissue. For each gene, the model was specified as:

$$Y_{i}^{ref}\;|\;ni∼Beta-Binomial\left(n_{i},\mu_{i},\theta \right),logit\left(\mu_{i}\right)=\beta_{0}+\beta_{1}\;.\;BC$$

Where $Y_{i}^{\text{ref}}$ and n_i_ is the number of reference allele reads, and total allele-specific reads for an individual i, respectively; μ_i_ is the expected reference allele proportion, Ө is the overdispersion parameter, and BC indicates the group (F_1_ × GL or F_1_ × Pi). The coefficient β_1_ measures the extent to which the expression of the reference allele differs between the two backcross groups, capturing the dASE effect. To quantify this effect size, a log2 fold change (log2FC) in reference allele proportion was calculated between groups as:

$$\log\;2\text{FC}=\log\;2\;\left(\frac{\mu \text{GL}+10^{-3}\;}{\mu \text{Pi}+10^{-3}}\right)$$

Where μGL and μPi are the mean reference allele ratios (ref/[ref + alt]) in F_1_ × GL and F_1_ × Pi samples, respectively, a pseudocount of (10^-3^) was added to both means to stabilize fold change estimates, particularly in cases of low expression or extreme allelic imbalance. Likelihood ratio tests were applied to evaluate group effects, and resulting *p*-values were adjusted using the Benjamini–Hochberg method to control the false discovery rate (FDR). Genes were considered to exhibit significant dASE if FDR <0.05 and |log2FC| > log2 (1.1). Significant dASE genes were visualized using the pheatmap package (version 1.02.12) within the R programming environment.

We further performed a post-hoc power analysis under the observed sample sizes for each tissue. In non-gonadal tissues (*n* = 12 per group), power exceeded 80% at an effect size of Δ ≈ 0.25, while in the ovary (9 vs. 8) and testis (10 vs. 8), this threshold was reached at approximately Δ = 0.25 and Δ = 0.30, respectively. These results indicate that our study was adequately powered to detect moderate-to-large dASE effects, but had limited sensitivity for small-effect variants.

#### Functional enrichment analysis

Functional enrichment was carried out using ClueGO (version 2.5.10)^96^ and CluePedia (version 1.5.10)^97^ within the Cytoscape (version 3.10.2)^98^ environment to identify significantly over-represented Gene Ontology Biological Process (BP) terms and KEGG pathways. Firstly, enrichment analysis was performed for global differential expression, comparing F_1_ × GL and F_1_ × Pi across all six tissues. Differentially expressed genes were divided into two clusters: Cluster 1 included genes with higher expression in F_1_ × GL than F_1_ × Pi, while Cluster 2 included genes with lower expression in F_1_ × GL than F_1_ × Pi. The results were visualized by stacked bar plots for GO BP using the ggplot2 (version 2.5.10; R package),^99^ and network-style diagrams for KEGG pathways within Cytoscape. Afterward, enrichment analysis was conducted on tissue-specific differential expression analyses, comparing expression between backcross groups within each of the six fetal tissues. A similar clustering strategy was used for tissue-specific enrichment results, with Cluster 1 containing genes (F_1_ × GL > F_1_ × Pi) and Cluster 2 containing genes (F_1_ × GL < F_1_ × Pi), and enrichment results were visualized as pie chart–style dot plots for GO BP and chord diagrams for KEGG pathway in ggplot2. Finally, the functional enrichment analysis was performed on tissue-specific identified ASE genes from F_1_ × GL and F_1_ × Pi BC groups. The ClueGO clustering of ASE genes produced functional groups in which Cluster 1 was composed of ASE genes from the F_1_ × GL backcross and Cluster 2 was composed of ASE genes from the F_1_ × Pi backcross. ASE enrichment results were visualized using pie chart–style dot plots for both GO BP and KEGG pathways in ggplot2. For all analyses, enrichment testing was performed using the hypergeometric test with Benjamini–Hochberg correction for multiple testing, and terms with adjusted *p* < 0.05 were considered significant.

#### Analysis of fetal weight distribution and genotypic clustering

Fetal body weights were recorded at gestational day 63 for 38 fetuses used for non-gonadal and gonadal tissues. Summary statistics (mean ± standard deviation) were calculated for each backcross group (F_1_ × GL and F_1_ × Pi). The significance of weight differences between backcross groups was assessed for each set (non-gonadal, ovaries, testes) using a two-sided Student’s *t* test in the R programming environment. Furthermore, to confirm the genetic divergence intrinsic to the reciprocal backcross design, we performed PCA on genome-wide SNP chip data from all 38 fetuses. A rigorously filtered genotype matrix was constructed by first excluding SNPs and individual samples with a missing genotype rate >20%. Remaining missing genotypes were then imputed to the mean allele dosage for each respective SNP. Genetic separation between backcross groups was visualized via PCA performed on the imputed genotype matrix. The statistical significance of this genetic separation was evaluated using a permutation-based multivariate analysis of variance (PERMANOVA) with 999 permutations, based on a Euclidean distance matrix and implemented in the vegan package (version 2.7.1) within R.^93^

#### Genomic overlap of ASE genes with pig QTL intervals and permutation testing

To functionally interpret ASE genes within established phenotypic frameworks, ASE genes identified across six tissues (brain, kidney, liver, muscle, ovary, and testis) and two backcross groups (F_1_ × GL and F_1_ × Pi) were mapped to pig quantitative trait loci (QTLs). Pig QTL data were obtained from the Animal QTL Database (QTLdb; Release 58, December 29, 2025; https://www.animalgenome.org/cgi-bin/QTLdb/SS/index).^105^ QTL intervals were classified into six phenotypic categories: growth, meat quality, reproduction, immune, feed efficiency, and carcass, using systematic keyword matching of trait descriptions. ASE gene coordinates were derived from Ensembl annotations (Sus-scrofa.Sscrofa11.1.100.gtf; Ensembl release 113) and converted to genomic ranges using the GenomicRanges package (version 1.52.1)^94^ within R.Genomic overlaps between ASE gene loci and QTL intervals were identified for each tissue–backcross combination. Because QTL intervals can be large in some studies, we first summarized their size distribution by calculating the median, mean, IQR, minimum, and maximum interval width per category. To test whether observed overlaps exceeded chance expectation, we implemented a permutation-based null model. For each tissue–backcross group, ASE gene positions were randomly shuffled across the genome 10,000 times, preserving chromosome distribution and gene length. For each permutation, the number of shuffled genes overlapping any QTL interval was counted, and an empirical *p*-value was calculated as the proportion of permutations in which the shuffled overlap count was greater than or equal to the observed count. The spatial distribution of ASE genes relative to QTL phenotypic categories was visualized using circos plots generated with the circlize package (version 0.4.15).^95^

## Supplemental information

Table S1. Summary of RNA-seq read mapping statistics across all samples

Table S2. Global differentially expressed genes between F1×GL and F1×Pi across all tissues

Table S3. Functional enrichment of global DEGs

Table S4. Tissue-specific differentially expressed genes

Table S5. Tissue-specific functional enrichment of DEGs

Table S6. Overlap of ASE genes with *cis*-eQTLs

Table S7. Functional enrichment of tissue-specific ASE genes

Table S8. Differential ASE genes between backcross groups

Table S9. QTL interval size distribution and permutation test results

Table S10. ASE gene-QTL overlap mapping
